# Realization of the Paris Agreement depends on adherence to the original spirit

**DOI:** 10.1093/nsr/nwab215

**Published:** 2021-11-29

**Authors:** Zhongli Ding

**Affiliations:** Academician of the Chinese Academy of Sciences, and Chief Scientist of the CAS Climate Change Research Group

Since the signing of the Kyoto Protocol in 1997, the road towards international cooperation in responding to global warming has been quite bumpy. Following the aborted Copenhagen Agreement, the Paris Agreement in 2015 strenuously reunited various countries based on the principle of individualized contributions. Yet, in the recent UN Framework Convention on Climate Change 26th summit meeting (COP26) held in Glasgow, US government leaders again pointed the finger at China, accusing China of not doing enough and setting insufficient goals for reducing carbon emissions. However, the US has only symbolically signed the Kyoto Protocol—having never obtained the approval of Congress—and has treated the Paris Agreement with a wavering position, seemingly forgetting the fact that the US is leading the world in various indicators of carbon emissions and, according to its commitment, future emissions will remain high. For this, we have to let the data speak.

It is well known that the increase of atmospheric CO_2_ concentration, from 280 ppm before the Industrial Revolution to the present level of 410 ppm, was due primarily to the use of fossil fuel. Over this period, advanced countries accomplished industrialization, urbanization and construction of basic infrastructure, and elevated their social welfare. Through these processes, advanced countries have in fact largely occupied the carbon emission space that could be available for later-developing countries. In discussing issues such as the responsibility to reduce carbon emissions in a scientific manner, two important factors of emission history and population size are inevitably involved.

To address this issue, we have calculated the cumulative total and per capita amount of carbon emissions of various countries during 1850–2019, based on existing authoritative data around the world. Over this period, the entire world emitted 1610 billion tons of CO_2_, of which the US contributed 410 billion tons, ∼25.5%. Despite having a population several times that of the US, China's total emission was only 220 billion tons, ∼13.7%, and India, Brazil and South Africa emitted 49.2, 14.6 and 18.5 billion tons (3.1%, 0.9% and 1.1%), respectively. Over this period, the average per capita amount for the entire world was 386 tons. It was 47, 92 and 490 tons for India, Brazil and South Africa, respectively, and 182 tons for China (only 47.2% of the world average). By contrast, it was 2174 tons in the US—12 times that of China. One wonders where US politicians get the courage to accuse China. The US promises a 52% reduction in carbon emissions by 2030 relative to that in 2005—from 6.13 to 2.94 billion tons per year. Even so, the per capita emission of an American will still be as high as 8.97 tons/year, nearly twice the current world average per capita (4.58 tons/year).

We hope US politicians will make a sincere return to the original principle of ‘common but differentiated responsibility’ of the Kyoto Protocol on global collaboration in dealing with climate change, a principle that was later reiterated in the Paris Agreement. The understanding of this principle by the international community has always been clear: the increase in atmospheric CO_2_ concentration was mainly caused by advanced industrial countries, which not only have the responsibility of a large-scale reduction of emissions but also the obligation to provide technological and financial support to help later-developing industrial countries to build a low-carbon environment. It was precisely based on this principle that many developing countries, including those that are mainly agricultural, signed the Kyoto Protocol. But developed countries did not keep their promise to provide realistic help, and continued to use various reasons and excuses to pressure developing countries into taking on unrealistic responsibilities.

China has responded to calls for international cooperation by addressing the issue of climate change sincerely and strenuously, as evidenced by major national efforts in promoting non-carbon energy sources in recent years. If it were merely using carbon-reduction promises as a geopolitical weapon, China could make a simple declaration that should silence all US proclamations: from 1850 to 2050, China's per capita carbon emissions have been and will be controlled at a level below one-quarter those of the US, regardless of how the US tries to reduce emissions!

We sincerely hope that US politicians return to the original spirit of international cooperation with regard to climate change, and avoid solidifying the ethically dangerous and huge rich–poor gap existing in the world. We also hope the US can quickly reach domestic consensus on how to deal with climate change, and stop the continuing farce of ‘democrats in, republicans out’ over the past two decades.

